# Dynamic NIR Fluorescence Imaging and Machine Learning Framework for Stratifying High vs. Low Notch-Dll4 Expressing Host Microenvironment in Triple-Negative Breast Cancer

**DOI:** 10.3390/cancers15051460

**Published:** 2023-02-25

**Authors:** Shayan Shafiee, Jaidip Jagtap, Mykhaylo Zayats, Jonathan Epperlein, Anjishnu Banerjee, Aron Geurts, Michael Flister, Sergiy Zhuk, Amit Joshi

**Affiliations:** 1 Department of Biomedical Engineering, Marquette University and Medical College of Wisconsin, Milwaukee, WI 53226, USA; 2 Department of Radiology, Mayo Clinic, Rochester, MN 55905, USA; 3 IBM Research Europe, D15 HN66 Dublin, Ireland; 4 Division of Biostatistics, Medical College of Wisconsin, Milwaukee, WI 53226, USA; 5 Department of Physiology, Medical College of Wisconsin, Milwaukee, WI 53226, USA

**Keywords:** cancer, tumor microenvironment modifier, notch-DLL4, consomic xenograft model, machine learning, binary classification, dynamic enhanced NIR imaging, indocyanine green, time series, tumor detection

## Abstract

**Simple Summary:**

Breast cancer is a disease that is affected by both the tumor cells and the host environment. It is well known that the tumor blood vessels are aberrant in structure and function due to rapid angiogenesis, and this aberrant vasculature plays a major role in drug delivery and therapy response of breast cancer. Dll4 is a protein that helps control the growth of blood vessels in tumors. This study used near-infrared optical imaging and a novel machine learning framework to determine if Dll4 levels can be predicted from simple noninvasive imaging assays. The eventual results of this study may help physicians decide if a given triple-negative breast cancer patient will benefit from a Dll4 targeted therapy.

**Abstract:**

Delta like canonical notch ligand 4 (Dll4) expression levels in tumors are known to affect the efficacy of cancer therapies. This study aimed to develop a model to predict Dll4 expression levels in tumors using dynamic enhanced near-infrared (NIR) imaging with indocyanine green (ICG). Two rat-based consomic xenograft (CXM) strains of breast cancer with different Dll4 expression levels and eight congenic xenograft strains were studied. Principal component analysis (PCA) was used to visualize and segment tumors, and modified PCA techniques identified and analyzed tumor and normal regions of interest (ROIs). The average NIR intensity for each ROI was calculated from pixel brightness at each time interval, yielding easily interpretable features including the slope of initial ICG uptake, time to peak perfusion, and rate of ICG intensity change after reaching half-maximum intensity. Machine learning algorithms were applied to select discriminative features for classification, and model performance was evaluated with a confusion matrix, receiver operating characteristic curve, and area under the curve. The selected machine learning methods accurately identified host Dll4 expression alterations with sensitivity and specificity above 90%. This may enable stratification of patients for Dll4 targeted therapies. NIR imaging with ICG can noninvasively assess Dll4 expression levels in tumors and aid in effective decision making for cancer therapy.

## 1. Introduction

Breast cancer heterogeneity has been extensively studied and it has enabled the classification and categorization of tumors into molecular subtypes depending on the overexpression of antigens or hormone receptors on tumor cells [1]. Identification of tumor subtypes improves cancer patients’ care and prognosis by tailoring therapies to the subtypes [2,3,4]. Breast and many other cancers are highly heritable, yet most causative variants are unknown, and most of the known risk variants are considered tumor-cell-autonomous, with far less emphasis placed on identifying the role of germline variants impacting the tumor microenvironment (TME). The TME is a complex and dynamic system that includes cancer cells, stromal cells, blood vessels, and extracellular matrix [5] and plays a significant role both in tumor cell proliferation and in chemo- or radiotherapy delivery and response [5,6,7]. There is growing evidence that heritable modifiers of the tumor microenvironment can profoundly impact tumor behavior and response to diagnostic and therapeutic interventions [8,9,10,11,12].

Tumor blood vessels have abnormal structure and function, which leads to heterogeneity in blood perfusion both temporally and spatially [13]. This heterogeneity has multiple adverse consequences, including limiting the access of blood-borne drugs and effector immune cells to poorly perfused regions of tumors [14]. As a result, these areas become hypoxic and have low extracellular pH [15]. Hypoxia has been shown to play a significant role in tumor progression and metastasis by inducing genetic instability, angiogenesis, immunosuppression, inflammation, and resistance to cell death by apoptosis and autophagy [16,17]. Anti-angiogenic drugs are designed to target the vasculature in order to starve tumors and prevent them from growing. However, recent studies have shown that the efficacy of these drugs may be limited by specific biomarkers and pathways associated with resistance [15]. For example, it has been shown that some patients may not benefit from anti-VEGF therapies if they have elevated levels of plasma sVEGFR1 [18]. Similar outcomes have been observed with increased levels of SDF1α and anti-VEGF therapies [19]. Further the vascular TME and therapy response differs in primary tumor and metastasis sites [20] and the anatomic location [21,22,23]. Thus, characterizing angiogenesis in tumors holistically may have therapeutic implications.

The process of tumor angiogenesis is closely regulated by a balance between promoting and suppressing angiogenic factors [24,25]. Delta like canonical notch ligand 4 (Dll4) is a protein-coding gene that provides instructions for making a protein part of a signaling pathway known as the notch pathway, which is essential for the normal development of many tissues throughout the body, affecting cell functions [26,27], modulating tumor angiogenesis [28], promoting vessel maturation, and inhibiting vessel sprouting by inducing apoptosis of tip endothelial cells (TECs) [28,29,30]. Dll4 is overexpressed in various types of cancer, including breast, ovarian, and colorectal cancer, and has been shown to promote tumor angiogenesis, growth, and metastasis by interacting with receptors on endothelial cells (ECs) [31,32,33,34]. Blockade of Dll4 activity results in enhanced vessel sprouting and increased vascular permeability [29,30,35], but anti-Dll4 therapy has not been universally successful, as Dll4 has been shown to have both pro-tumorigenic and anti-tumorigenic effects depending on the context of its expression [34,36,37]. We recently reported that Dll4 expression on the host TME rather than on tumor cells determines the EPR or enhanced permeation and retention effect in breast tumor xenografts and thus governs nanomedicine delivery and therapy response [38].

Despite the increasing evidence about the function of germline genetic modifiers, such as Dll4, in TME heterogeneity and enhanced permeability and retention (EPR) effects, the underlying influencers have mainly remained unexplored because of the lack of a systematic approach to studying them. Therefore, we developed the Consomic Xenograft Model (CXM) as a strategy for mapping heritable modifiers of TME heterogeneity. In the CXM, human breast cancer cells are orthotopically implanted into consomic xenograft host strains. These strains are derived from two parental strains with different susceptibilities to breast cancer. Salt-sensitive (SS) rats were employed as a tumor promoting strain, while Brown Norway (BN) rats were used as a tumor suppressing strain. A sequence of consomic strains were generated with chromosomes of SS rats replaced by those of BN rats one at a time and used for breast tumor xenograft studies [7,38,39]. Because the host backgrounds genetically differ by one chromosome, whereas the tumor cells are unvaried, any observed phenotypic differences are due to TME modifier(s) and can be linked to a single chromosome. These modifiers can be further localized by congenic mapping (inbred strains containing a given sub-chromosomal region in their genome). By combining CXM with dynamic epifluorescence near-infrared (DE-NIR) imaging, systemic injections of indocyanine green (ICG) through a tail vein in tumor-bearing rats, and multiparametric analysis of pharmacokinetic modeling, we localized and identified the function of the vascular-specific Dll4 allele on rat chromosome 3 (RNO3) as a heritable host TME modifier of EPR [38]. The SS.BN^3IL2Rγ−^ CXM strain with low-level expression of Dll4 (referred to as Dll4−) had significant tumor growth inhibition compared with the parental SS^IL2Rγ−^ strain with higher expression of Dll4 (Dll4+), despite a paradoxical increase in tumor blood vessel density in Dll4+. Further analysis revealed that the changes in the Dll4+ tumors were accompanied by altered expression of Dll4, which was previously linked with nonproductive angiogenesis. Additionally, Dll4 was found to be co-localized within a host TME modifier locus (Chr3: 95–131 Mb) identified by congenic mapping and correlated with the phenotypic differences observed at the consomic level [7,39,40].

The inheritance of functionally different Dll4 alleles can influence the efficacy of nanoparticle (NP) therapy, and previous results indicate that inherited microvascular distribution patterns, rather than overall NP uptake, ultimately determine the effectiveness of NP-mediated photothermal therapy (PTT). Consequently, patients with high endothelial Dll4 expression can be selected for treatment with anti-Dll4 targeted nanoparticles as opposed to patients with low Dll4 expression, where PEGylated nanoparticles will provide sufficient therapy response [38]. Recent advances in dynamic vascular imaging techniques, such as DCE-MRI and perfusion computed tomography, have facilitated the investigation of the time kinetics of a contrast agent to extract multiple vascular parameters and have been successfully applied in clinical trials of anti-angiogenic drugs [41,42]. However, these techniques have certain drawbacks, including a lack of high temporal resolution and the need for a heavy hardware system with sophisticated analysis software. Dynamic NIR fluorescence imaging, on the other hand, offers a sufficient and effective alternative to other dynamic vascular imaging techniques for characterizing germline-dependent vascular phenotypes [7,43]. This has led to the combination of these modalities, such as in the paired agent MRI-coupled fluorescence tomography approach for noninvasive quantification of paired-agent uptake in response to anti-angiogenesis therapy in vivo [44].

As the field of artificial intelligence continues to advance, researchers are increasingly utilizing AI techniques, particularly machine learning, to develop predictive models that can support effective decision making in various domains including cancer therapy selection [45,46]. Previous research has investigated the use of machine learning algorithms to analyze near-infrared (NIR) signal intensity and perfusion patterns to differentiate between healthy, benign, and malignant tissue [47]. This work demonstrated that the signal intensity time course of an FDA-cleared near-infrared dye ICG inflow during the wash-in phase and ICG outflow during the wash-out phase could serve as significant markers for tissue distinction. This finding offers a new method for noninvasive tissue distinction and has prognostic potential [48,49]. However, there remains a need for further exploration of the use of machine learning for classifying host genetic tumor microenvironment (TME) modifiers and predicting therapy responses based on dynamic contrast-enhanced imaging of tumors, particularly DE-NIR fluorescence imaging data [47].

## 2. Hypothesis and Objective

**Figure 1:**
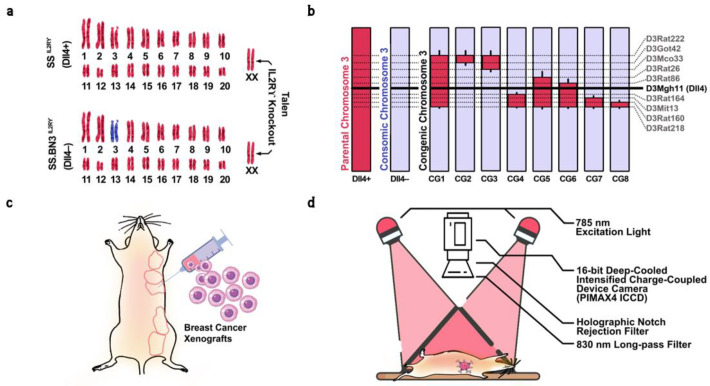
(**a**) Schematic representation of the SS(Dll4+) and SS.BN3(Dll4−) genomes modified by TALEN-mediated editing of the IL2Rγ gene, showing chromosomes derived from SS (red) or BN (blue). (**b**) Schematic representing congenic strains and vascular-specific Dll4 protein expression, generated by introgressing BN(Dll4−) chromosome 3 (blue) into the SS^IL2Rγ−^ strain (red) via marker-assisted breeding. Thin black bars represent confidence intervals of chromosomal regions that could be BN or SS. The horizontal black line indicates the location of vascular-specific Dll4 protein on chromosome. The dotted lines represent the location of SSLP markers. (**c**) Transgenically labeled human MDA-MB-231 triple-negative breast cancer cells were orthotopically implanted in the mammary fat pad of parental, consomic, and congenic rats. (**d**) Schematic diagram of the dynamic epifluorescence NIR imaging setup. A bifurcated optical fiber bundle delivers 785 nm excitation light for uniform illumination of the rat body surface. A 16-bit deep-cooled intensified charge-coupling device camera was used to image the rats through computer-controlled software.

We hypothesize that the observation of subtle differences in vasculature structure and perfusion patterns characterized by ICG inflow and outflow using DE-NIR imaging could be used to differentiate between inherited tumor vascular microenvironment differences, such as Dll4 expression levels. We propose an experimental framework to noninvasively assess Dll4 expression levels in tumors based on the NIR signal intensity time course of perfusion patterns characterized by ICG time kinetics to develop a predictive model to support effective decision making in cancer therapy. Herein, we used two rat-based CXM strains of breast cancer, SS^IL2Rγ−^(Dll4+) and SS.BN3^IL2Rγ−^ (Dll4−) [7,38,50], as well as eight congenic xenograft strains, CG1–CG8 (Figure 1a,b), to assess the impact of germline TME vascular heterogeneity on the signal intensity of DE-NIR imaging with systemically delivered ICG. Principal component analysis (PCA)-based decomposition of time-dependent epifluorescence videos (image stacks) was used for visualization and anatomical segmentation of tumors, liver, lungs, and fat pads [7]. In addition, we utilized modified principal component analysis (PCA)-based anatomical segmentation techniques to identify and analyze regions of interest (ROIs) representing potential tumors within the current dataset. To gather further information, we calculated the average NIR intensity for each ROI by analyzing the brightness of individual pixels at each time interval, resulting in a series of intensity measurements for each ROI. From this analysis, several easily interpretable features were extracted, including the slope of the initial uptake of ICG, the time it takes to reach peak perfusion, and the rate of ICG intensity changes once the half-maximum intensity is reached (which, to the best of our knowledge, has not been previously reported in the literature). We then applied a subset of machine learning algorithms, including Support Vector Machines (SVMs), Naive Bayesian Classifiers (NBCs), Generalized Additive Models (GAMs), Decision Trees (DTs), Nearest Neighbors (NN), and Logistic Regression (LR) to select the most discriminative features for classification. The performance of the model was evaluated using confusion matrix, receiver operating characteristic curve (ROC), and the area under the curve. To further evaluate our hypothesis of detecting Dll4 expression levels from DE-NIR imaging and test the generalizability of our framework, we conducted a secondary performance evaluation method using congenic groups with high and low Dll4 expression levels. The classification models were trained based on the selected features, and the performance of the model was tested on the remaining congenic groups. We demonstrate that robust ML methods can identify the alterations in host Dll4 expression from the tumor dynamic imaging datasets, and thus these methods can potentially stratify patients for Dll4 targeted therapies.

## 3. Materials and Methods

All methods have been carried out in accordance with relevant guidelines and regulations. Approved protocols by the Medical College of Wisconsin Institutional Biosafety Committee (IBC) and Institutional Animal Care and Use Committee (IACUC) were followed. All live animal experiments are reported per the ARRIVE guidelines’ recommendations [51]. All results were rigorously adjusted for multiple comparisons.

### 3.1. Animals

All animal protocols employed in this study were approved by the Institutional Animal Care and Use Committee (IACUC), Medical College of Wisconsin (MCW). The MCW has an Animal Welfare Assurance (Assurance number D16-00064 (A3102-01)) on file with the Office of Laboratory Animal Welfare, National Institutes of Health (NIH). Animal experiments were performed according to the relevant guidelines and regulations and in compliance with the Guide for the Care and Use of Laboratory Animals published by the US National Institutes of Health (NIH publication no.85–23, revised 1996). SS.BN3 rats were developed as part of the Consomic Xenograft Model at the Medical College of Wisconsin (MCW) [40]. SS and SS.BN3 rats were purchased from the Rat Research Models Service Center at the Medical College of Wisconsin [52]. All rats were provided reverse osmosis hyper-chlorinated water ad libitum. All animal experiments were performed on anesthetized animals. The animal was placed in a transparent induction chamber to induce anesthesia. Isoflurane was delivered through a precision vaporizer and compressed O_2_ to the chamber. For induction, the percentage of isoflurane was up to 5%. Once the animal was unconscious, it was removed from the chamber. The unconscious animal was then placed on a warm surface and fitted with a nose cone attached to the vaporizer in the presence of a scavenging system and oxygen source. At this point, the concentration of isoflurane was reduced to this level that maintained the correct anesthesia plane, usually between 0.5 and 3%. After the end of the experiment, or when other criteria for animal protocols were justified, rats were euthanized. Rats were placed in an approved euthanasia chamber and exposed to CO_2_ from a compressed gas cylinder until the animal was no longer breathing. To ensure death in rats, a pneumothorax was created via thoracotomy for rats weighing more than 200 g. For rats weighing less than 200 g, a pneumothorax was created, or a cervical dislocation was performed.

As previously described [39,40], consomic strains (SS and SS.BN3 rats) were generated by sequentially replacing SS chromosomes with the outbred wild-type and tumor-resistant strain of Brown Norway (BN) rats referred to as SS.BN#, which are reported for their tumorigenic potential, where # refers to the chromosome number. These parental SS and consomic SS.BN# strains were genetically ablated by knocking down the IL2Rγ gene to allow the grafting and growth of human cancer cell lines. Such immunocompromised strains are labeled as SS^IL2Rγ−^(Dll4+) and SS.BN#^IL2Rγ−^(Dll4−).

Previous research has localized inherited modifier(s) of TME vascular heterogeneity to RNO3 by CXM mapping and further narrowed by congenic mapping to a 36 Mb locus containing Dll4 alleles with distinct vascular expression patterns in the SS.BN3^IL2Rγ−^ consomic (Dll4−) and SS^IL2Rγ−^ (Dll4+) rat strains, and verified via species-specific RNA sequencing and immunohistochemistry that strains inheriting the SS Dll4 allele on chromosome 3 have higher Dll4 expression on tumor-associated endothelium and that the blood vessel tortuosity and dysfunction increased in Dll4− strains [38,39]. Since there are many other candidate alleles on chromosome 3 that could also potentially account for the observed differences in therapeutic efficacy between the SS.BN3^IL2Rγ−^ and SS^IL2Rγ−^ strains [38], to further investigate the potential contribution of Dll4 to inherited tumor vascular heterogeneity, eight novel SS.BN3^IL2Rγ−^ congenic xenograft host strains (CG1 to CG8) were constructed by introgression of the F1 progeny and F2 generation to capture different regions of RNO3 by marker-assisted selection, as described previously [50,53]. The exclusion congenic mapping localized a 7.9 Mb candidate region (marked by the SSLP marker D3Mgh11) that was associated with inherited tumor vascular heterogeneity and contained the Dll4 locus. As a result, eight new congenic strains CGN(s-e) were generated, where N (1 to 8) refers to the congenic group, while s and e refer to the starting and ending of Simple Sequence Length Polymorphism (SSLP) marker regions, respectively. This resulted in generating CG1(D3Rat26-D3Mgh30), CG2(D3Rat222-D3Got42), CG3(D3Rat222-D3Mco33), CG4(D3Rat164-D3Rat218), CG5(D3Rat26-D3Mco218), CG6(D3Rat86-D3Rat218), CG7(D3Mgh13-D3Rat218), and CG8(D3Rat160-D3Rat218) congenic groups (Figure 1a,b).

### 3.2. Cell Culture and Triple-Negative Breast Cancer Xenografts

As previously described [38], triple-negative breast cancer MDA-MB-231 cells were maintained in DMEM media (Sigma, Burlington, MA, USA) supplemented with 10% FBS (Gibco, New York, NY, USA) and 1% penicillin and streptomycin (Lonza, Cohasset, MN, USA) and incubated in 5% CO_2_ at 37 °C. These cells (6 × 10^6^) in 50% Matrigel were orthotopically implanted into the mammary fat pads (MFP) of 4- to 6-week-old female Dll4+ (*n* = 8) and Dll4– (*n* = 17) rats and eight congenic strains CG1 (*n* = 19), CG2 (*n* = 2), CG3 (*n* = 26), CG4 (*n* = 12), CG5 (*n* = 28), CG6 (*n* = 12), CG7 (*n* = 5), and CG8 (*n* = 4) (Figure 1c) [54]. Tumors were treated after 10 days of implantation at an approximate size of 600 mm^3^, consistent across all rat strains.

### 3.3. In Vivo NIR Fluorescence Imaging

A customized NIR imaging system was assembled for imaging the rats. A bifurcated optical fiber bundle was used to deliver 785 nm excitation light (~5 mW/cm^2^ power at the surface, diode laser, ThorLabs Inc., Newton, NJ, USA) from two positions for uniform illumination of the entire rat body surface. A 16-bit deep-cooled intensified charge-coupled device camera (PIMAX4 ICCD, Princeton Instruments, Trenton, NJ, USA) equipped with 830 nm long-pass filter positioned following a holographic notch rejection filter in the optical path (ThorLabs Inc.) was used to image the rats through computer-controlled LightField^®^ software (Teledyne Princeton Instruments, Trenton, NJ, USA) (Figure 1d).

Dynamic contrast-enhanced NIR fluorescence imaging was performed on anesthetized rats, as reported previously for 800 nm NIR imaging [7]. In this study, the setup was used for imaging the whole body. A total of 133 rats (Dll4+ (*n* = 8), Dll4– (*n* = 17)) and eight congenic strains CG1 (*n* = 19), CG2 (*n* = 2), CG3 (*n* = 26), CG4 (*n* = 12), CG5 (*n* = 28), CG6 (*n* = 12), CG7 (*n* = 5), and CG8 (*n* = 4) were imaged. NIR imaging was performed for approximately 6 min following ICG injection with the CCD array hardware binned to 256 × 256 with a frame rate of 10.6 fps. A total of 3000 frames were captured for each imaging case, including about 50 frames for background correction. ICG (MP Biomedicals) was delivered in an intravenous bolus of 1200 µM ICG/200 g body weight into the tail vein via a catheter with a 32-gauge needle tip connected to a syringe pump (Harvard Apparatus PHD 2000 syringe pump, Holliston, MA, USA) operated at a speed of 0.2 mL/s. The injected volume was calibrated to provide a body-weight-equilibrated dose to each rat.

### 3.4. Denoising and Motion Correction

Image processing and data analysis were performed in MATLAB (R2021b MATHWORKS Inc.) software. The time-dependent image frames were assembled as 3D arrays (two spatial and one temporal dimensions) for all animals. A custom-designed breathing correction method with a low-pass temporal filter combined with a 1D wavelet-based denoising was used to filter the high-frequency jitter generated by the animal’s respiratory motion from the fluorescence kinetic sequences of each pixel, as described previously [7]. An average of pre-ICG injection frames (acquired in the ~5 s before ICG injection) was used as background, incorporating contributions from CCD noise and excitation light leakage from emission filters and subtracted from all the frames. (Refer to Video S1 for respiratory motion corrected time course images of ICG biodistribution).

### 3.5. Principal Component Analysis for Extraction of Spatial Patterns of Internal Organs

First, motion correction and background subtraction were performed on the imaging data described in the previous steps. This was carried out to remove any potential artifacts or noise that could affect the accuracy of the principal component analysis. Next, the data were decomposed by PCA along the time dimension using MATLAB software following the previously published methods [55,56]. This resulted in converting the imaging data to a k-component vector for each pixel, where k is the number of time-frames in the original dataset. The PCA on the dynamic fluorescence image was used to extract spatial patterns of internal organs linked to statistically similar kinetic behaviors. This was carried out by comparing the k-component vectors for each pixel and identifying those that displayed similar patterns over time. The contribution of the first six principal components on a time basis is illustrated in Figure S1.

### 3.6. PCA Ranking Tumor Detection

**Figure 2:**
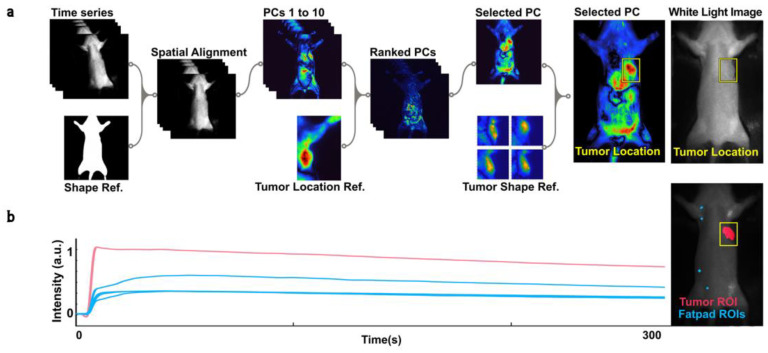
(**a**) Diagram of the process for detecting tumors and generating regions of interest (ROIs). The ROI detection module consists of three steps: spatial alignment, PCA ranking and selection, and ROI selection and masking. First, NIR time series data are registered to a reference image using a rigid body transformation. Next, PC reconstructions are ranked based on their 2D cross-correlation with a reference image featuring a clearly visible tumor. The tumor’s location and a bounding box (shown in yellow) are estimated based on the 2D cross-correlation score of the selected PC and reference images featuring different tumors. The tumor’s boundaries are enhanced and distinguished from the background through morphological dilation and thresholding. The resulting ROI is used to mask the NIR time series data. (**b**) Plot of the mean intensity of ICG biodistribution fluorescence kinetic data over 300 s for the tumor (red) and four fat pads (blue), normalized to a range of 0 to 1.

The ROI detection module employed three steps in order to identify the tumor area in images (Figure 2a): (1) spatial alignment, (2) PCA ranking and selection, and (3) ROI selection and masking. First, images were registered to a reference image using a rigid body transformation in order to ensure consistent spatial alignment for the detection of the tumor area in subsequent steps. The variability in the visual appearance of internal organs or tumors within certain principal components necessitated the adaptation of existing methods for ranking normalized components based on their two-dimensional cross-correlation (2DCC) with a reference image containing the tumor. This was achieved by generating a stack of the first ten normalized principal components and applying a two-dimensional cross-correlation function (xcorr2 in MATLAB) to each component. The principal components were ranked according to the correlation scores obtained and a semi-automatic algorithm was then utilized to carry out the subsequent two steps for tumor ROI generation.

To select the appropriate principal component for tumor identification, the algorithm prioritized the PC with the highest likelihood of containing the tumor tissue based on the ranking from the previous step. Once the proper PC was selected, we used 2DCC to estimate the tumor’s location in the frame. This was performed by creating a stack of four reference images using PCs that clearly visualized the tumor and generated a bounding box around the tumor. Next, we applied basic morphological dilation and image arithmetic operations to emphasize the tumor’s boundaries. A threshold was then applied to separate the tumor from the background. Finally, we used Blob Analysis (Computer Vision Toolbox™, MATLAB, MathWorks, MA, USA) to extract the centroid and exact location of the tumor in the frame, generating a region of interest (ROI). The use of a graphic user-interface-enabled semi-automatic platform allowed for real-time evaluation and adjustment of the algorithm’s performance if necessary.

### 3.7. Video Processing

The underlying assumption for the NIR fluorescence intensity video analysis is that intensity was proportional to the ICG concentration in the tissue, which has been shown in the literature [57].

From each available video, p, between 4 and 5 fluorescence time series are extracted, one based on the tumor ROI generated from earlier steps, and between three and four from mammary fat pads (MFPs) (Figure 2b). For each ROI, r, and each included time step, t, the mean brightness of the pixels inside that ROI in the NIR video is stored as $I_{p,r}\left(t\right)$. The result is a collection of time series $I_{p,r}\left(t\right)$, where p ranges from 1 to N (the number of animals in each group in our dataset), r ranges from 4 to the number of ROIs generated in the specific animal (up to 5, 1 tumor and 3 or 4 fat pads), and *t* from 0 to 3000 (Figure 2b).

### 3.8. Peak and Latency Estimation

First, we started with smoothing the data to exclude any potential noise from motion artifacts. The smoothing was conducted using a Savitzky–Golay filter of order 3 with window length 31 [58]. The parameters for this filter were determined through manual annotation of peak and latency in a subset of the data, with the goal of minimizing average estimation error. Subsequently, we used a MATLAB function to identify the time point of maximum intensity, which corresponded to the peak of the fluorescence signal. We employed a custom latency detector script that analyzed the smoothed derivative of the curves and identified the first “robust” zero crossing as the latency point ($L_{p,r}\left(t\right)$) [48].

### 3.9. Feature Design

**Figure 3:**
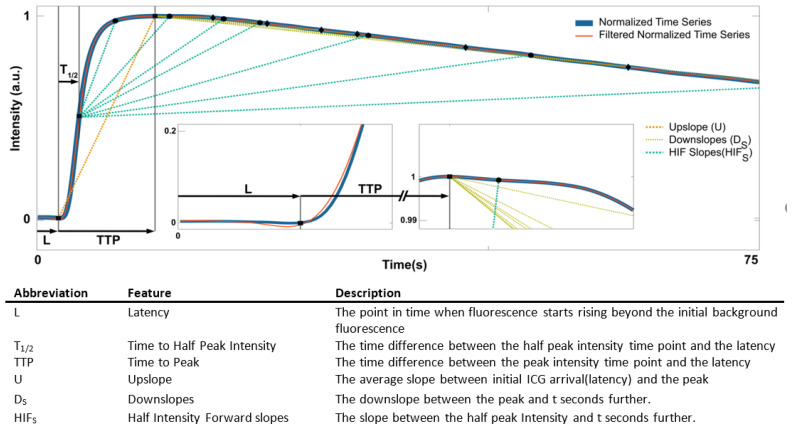
Feature extraction. The normalized mean intensity of ICG taken over ROIs is plotted for the first 75 s (blue). The time series was smoothed using a Savitzky–Golay filter of order 3 with window length 31 (red). The insets show enlarged curve regions to visualize the characteristics and relationships of the features. S = {2, 4, 5, 6, 8, 10, 12, 14, 16, 18, 20, 23, 25, 30, 35, 40, 50, 60, 70, 80}.

The features were chosen in two steps as previously described (Figure 3) [47,48]: first, the following characteristic numbers were chosen for each normalized time series individually.

The time to peak ($TTP_{p,r}$) is simply the time difference between the peak intensity time point and the latency $L_{p,r}$.

The upslope ($U_{p,r}$) is computed as

(1) $$U_{p,r}=1-\frac{I_{p,r}\left(L_{p,r}\right)}{TTP_{p,r}},$$

which is the average slope between initial ICG arrival and the peak.

The downslopes (DS) are the downslopes between the peak and S seconds further:

(2) $$D_{p,r}=1-\frac{I_{p,r}\left(L_{p,r}+TTP_{p,r}+S\right)}{S},\;S\in \left\{2,4,5,6,8,10,12,14,16,18,20,23,25,30,35,40,50,60,70,80\right\}.$$

The time ratio (TR) is the ratio between $TTP_{p}$ and when $I_{p}\left(t\right)$ reaches half the peak values.

The half intensity forward (HIF) is the average slopes between when $I_{p,r}\left(t\right)$ reaches half the peak values ($T_{1/2p,r}$) and S seconds further, so

(3) $$HIF_{p,r}=1-\frac{I_{p,r}\left(L_{p,r}+T_{1/2p,r}+S\right)}{I},\;S\in \left\{2,4,5,6,8,10,12,14,16,18,20,23,25,30,35,40,50,60,70,80\right\}.\;$$

To increase the robustness of estimated downslope-based and HIF-based features, we introduced a window around $L_{p,r}+TTP_{p,r}+S$ and $L_{p,r}+T_{1/2p,r}+S$ time steps taking the median of those downslopes:

(4) $$DS_{p,r,avg}=median\;s\in Window\;D{\left(S+s\right)}_{p,r},$$

(5) $$HIF_{p,r,avg}=median\;s\in Window\;D{\left(S+s\right)}_{p,r},\;Window=\left\{-1.5,-1.3,\ldots ,1.3,1.5\right\}\mathrm{seconds}.$$

These features provide insight into the tumor’s ICG uptake and decay. U and TTP relate to the initial uptake of ICG, while DS relates to the decay of ICG fluorescence [48]. TR is a measure of the temporal inhomogeneity of the initial uptake, and HIF is a measure of the temporal inhomogeneity of both the initial uptake and decay of ICG fluorescence.

To address inter-animal variation, we propose a feature design that relates the features of tumor intensity ($F_{r=tumor}$) to the features of healthy tissue intensity ($F_{r=fatpad}$) in the same animal. The median value of a feature across the healthy tissue (fat pad) is chosen as a reference value, and each feature is defined as its percentage difference to that reference value. This results in a single normalized feature for each animal. By using the median as an average that is robust to outliers, our feature design allows for a more accurate comparison of tumor intensity across different animals.

(6) $$F_{n\;}=\frac{F_{tumor}-median\left(F_{fat\;pad}\right)}{median\left(F_{fat\;pad}\right)},$$

### 3.10. Classification Algorithms and Feature Selection

The feature extraction and design required specialized knowledge of the tumor microenvironment (TME) and its impact on near-infrared intensity. However, the classification based on the inheritances of Dll4 can be considered a standard binary classification problem with feature selection as a sub-problem: given the features of an animal for which the Dll4 inheritance is unknown, we want to assign the label “Dll4high” or “Dll4low” to it. We also want to investigate which small subset of features performs best [44]. We restricted ourselves to a subset of available ML algorithms which were reported to perform best with intensity time series classification. [48], We excluded neural networks from consideration and evaluated Support Vector Machines (SVMs), Naive Bayesian Classifiers (NBs), Generalized Additive Models (GAMs), Decision Trees (DTs), Nearest Neighbors (NN), and Logistic Regression (LR).

### 3.11. Primary Classification

DTs and SVMs using the full set of 86 features were trained. Each ML model was tuned to the training set in an internal cross-validation procedure of 10-fold. This process was repeated 20 times, and the performance metrics are reported as classifier performance. One of the key metrics used to evaluate the performance of a classification algorithm is accuracy, which measures the proportion of correctly classified instances in the dataset. Other important metrics include sensitivity, which measures the proportion of positive instances that were correctly classified, and specificity, which measures the proportion of negative instances that were correctly classified. In our case, we are interested in classifying animals as either Dll4high or Dll4low, and we use the average metric (*A_score_*) for each pair of groups to evaluate the performance of the different classification algorithms.

The *A_score_* for a given pair of groups is calculated as the average of accuracy, sensitivity, and specificity, as follows:

(7) $$A_{score}\;(Dll4^{high}|\;Dll4^{low})=\frac{Accuracy+Sensitivity+Specificity}{3},$$

### 3.12. Congenic Pair Selection

In this study, we describe a two-step method for selecting congenic pairs with high and low Dll4 expression levels for feature selection and hypothesis testing. This method was used to identify well-behaved pairs for binary classification and to identify the pair with the highest classification performance: The primary selection of congenic pairs is based on their primary classification scores ($A_{score}$) and their performance against parental strains using all available features. The secondary selection is based on the classification performance using the 2 best-performing features.

The congenic pair selection process involved the selection of all possible combinations of congenic pairs with high and low Dll4 expression levels with *n* > 5, resulting in a total of 12 pairs: Dll4+|Dll4−, Dll4+|CG3−, Dll4+|CG4−, Dll4−|CG1+, Dll4−|CG5+, Dll4−|CG6−, CG1+|CG3−, CG1+|CG4−, CG3−|CG5+, CG3−|CG6+, CG4−|CG5+, CG4−|CG6+. It should be noted that we only included subgroups with *n* > 5 in our analysis, to avoid introducing noise into the feature selection process. This is because smaller sample sizes can be more prone to variability and may not represent the larger population [59]. Therefore, we focused on more significant subgroups to ensure our feature selection process was robust and reliable. These 12 pairs have gone through our primary classification algorithm with a split of 75%–25% for testing and training with seeded randomization and proportional distribution of each group in training and testing datasets. Each ML model was tuned to the training set in an internal cross-validation procedure of 10-fold and evaluated by its performance on the test set. This process was repeated 20 times, and the best *A_score_* was reported as the metric of classifier performance. The result of this step was used to identify well-behaved congenic pairs for binary classification by calculating the separation score (*S_score_*) for each pair of congenic groups with CG#+ and CG#− as below, which is bound between 0 and 1 and reported in Table 1:

(8) $$S_{score}\;(\mathrm{CG}\#+|\;\mathrm{CG}\#-)=\frac{A_{score}\;(Dll4+|\;\mathrm{CG}\#-)+A_{score}\;(\mathrm{CG}\#+|\;Dll4-)+2*\;A_{score}\;(\mathrm{CG}\#+|\;\mathrm{CG}\#-)}{4},$$

The congenic pairs with *S_score_* above 80% were selected for secondary congenic pair selection.

For the secondary selection, we evaluated the classification performance ($A_{score}$) of each pair determined this time by using the two most effective features as described in Section 3.13. We used a 75%–25% split for testing and training, with randomization to ensure a proportionate representation of each group in both datasets.

The pair with the highest classification performance, *A_score_*, based on the two most effective features was selected for the final classification model training. To ensure a high-quality classification model, we set a minimum threshold for the *A_score_* of 0.70 for inclusion in the feature selection process. Congenic groups with an *A_score_* below 0.70 were considered to have an insignificant contribution to the classification model and were excluded from further consideration in the feature selection process. This approach allowed us to focus on the most informative feature pairs, improving the overall classification performance of our machine learning model. Each machine learning model was tuned to the training set using a 10-fold cross-validation procedure and evaluated based on its performance on the test set. We repeated this process 20 times and reported the best *A_score_* as the classifier’s performance.

### 3.13. Feature Selection and Secondary Classification

We selected the best pair of features in terms of achieved sensitivity, specificity, and accuracy by a two-step procedure as previously reported: DTs and SVMs using the full set of 86 features were trained, and recursive feature elimination (RFE) was performed to refine a much smaller set of best-performing features [60]. RFE is a widely used machine learning classification algorithm that helps in reducing the dimensionality of feature space and selecting a small subset of features that yield the best classification performance. This was achieved through an iterative procedure that uses a ranking criterion to eliminate features one or more at a time. The RFE algorithm started by selecting a subset of features and training a model on this subset. The features were then ranked based on their contribution to the model’s performance, and the least important feature was eliminated. The process was then repeated with the remaining features, and the best subset of features was selected based on a model selection criterion [61].

One of the main advantages of RFE is that it helps to reduce the risk of overfitting when the number of features is large, and the number of training patterns is comparatively small [62]. This is because the algorithm selects only a subset of features that are relevant to the classification task, and this helps to avoid the inclusion of irrelevant and redundant features. RFE can be used in conjunction with other techniques such as regularization and support vector machines (SVMs) to further improve the performance of the classification model. In addition, projection methods such as principal component analysis can reduce the feature space’s dimensionality before applying RFE [55].

We used a k-fold cross-validation strategy to assess the performance of our model. We also reserved a portion of the training data for primary testing of the model after hyperparameter optimization. Our experiments were conducted with 20 random splits of the training and test datasets, and the mean performance metrics were reported for sensitivity, specificity, and accuracy as *A_score_*. To facilitate the interpretation of our results, we limited the number of final features to two. Furthermore, given the small size of our dataset, there was no justification for using high-dimensional feature spaces.

### 3.14. Data Augmentation

The use of data augmentation has become a popular technique in machine learning and deep learning, especially in the field of computer vision. Data augmentation involves applying random transformations to the training dataset to increase its diversity and improve the performance of a model. In this study, we used data augmentation on raw near-infrared (NIR) image stacks to evaluate the robustness of a classification model. We used a dataset of 3000 frames of the original raw 256 × 256 NIR images for this part of our study. These images were augmented using TensorFlow and the Keras API, which allowed us to apply random transformations to the dataset. The transformations included random rotation followed by a horizontal flipping, and up to 2% rescaling.

### 3.15. Training and Testing Dataset

The final training and testing dataset for the machine learning models was determined by the outcome of the congenic pair selection and feature selection steps. This dataset included all congenic groups except for Dll4+ and Dll4−, as well as the selected CG#+ and CG#− groups in the previous step. This step was conducted separately for the original dataset and augmented dataset. The models were trained using 10-fold cross-validation and a portion of the training data was reserved for testing after hyperparameter optimization, with 25% for the original dataset and 20% for the augmented dataset. The performance of the models was assessed using a confusion matrix, receiver operating characteristic curve (ROC), and the area under the curve.

### 3.16. Statistical Analysis

Repeated measures models are a powerful tool in statistical analysis that allow researchers to study the effects of different factors on a given outcome while accounting for the inherent dependence of multiple measurements taken on the same subject. In this study, a mixed effects model with appropriate time varying covariates was used to analyze the average fluorescence intensity of indocyanine green (ICG) in the tumor with multiple measurements per subject, with the subject number serving as the repeated measure indicator and the rat strain serving as a covariate. This allows for flexible time-based modeling when using multiple measures, likely dependent from the same animal [63,64]. Customized scripts in MATLAB were used to generate the fitted coefficients, covariance parameters, design matrix, error degrees of freedom, and between- and within-subjects factor names for the repeated measures model. The output was then analyzed with a multiple comparison of the estimated marginal means based on the variable strain, using the Tukey–Kramer test statistic [65]. This allowed estimation of multiplicity-adjusted *p*-values for the post hoc comparisons, which indicate whether the groups significantly differed with respect to strain. The data were then visualized as a *p*-value matrix, providing a clear illustration of the significant differences between groups.

### 3.17. Data Availability

This study employed the established consomic rat models SS and SS.BN3 as well as our congenic strains CG1 to CG8. The publicly accessible and NIH-supported Rat Genome Database (rgd.mcw.edu) catalogs has tools to explore the genotype and phenotype information for the SS (Dll4+) and SS.BN3 (Dll4−) and congenic strains under strain records: Dll4+ (RGDID:61499), Dll4− (RGDID:1358154), CG1 (RGDID:155782881), CG2 (RGDID:155782883), CG3 (RGDID:155782884), CG4 (RGDID:155791428), CG5 (RGDID:155791426), CG6 (RGDID:155791430), CG7 (RGDID:155791429), and CG8 (RGDID:155791427).

## 4. Results and Discussion

### 4.1. Dynamic Contrast-Enhanced NIR Fluorescence Imaging and Tumor Detection

Dynamic contrast-enhanced NIR fluorescence imaging has been widely used for tumor detection in various studies [66,67,68]. The use of NIR imaging allows for the visualization of internal organs and tissues without the need for invasive procedures, which can be particularly useful in detecting tumors due to their vascular heterogeneity compared to surrounding healthy tissues.

In previous studies, the use of principal component analysis (PCA) on the time domain of dynamic fluorescence images was utilized to extract spatial patterns of internal organs linked to statistically similar kinetic behaviors, such as liver, kidneys, lungs, and various tumors [7,56]. However, this technique required manual inspection and selection of proper principal components, which was time consuming and prone to human error and bias.

In order to overcome the limitations present in the current dataset, we implemented a modified method that utilizes near-infrared imaging and principal component analysis to detect tumors with high accuracy and without the need for manual correction (Figure 2 and Figure S1). The use of principal component analysis in this context not only allows for dimensionality reduction and noise removal but also enhances the robustness and efficiency of the method.

**Figure 4:**
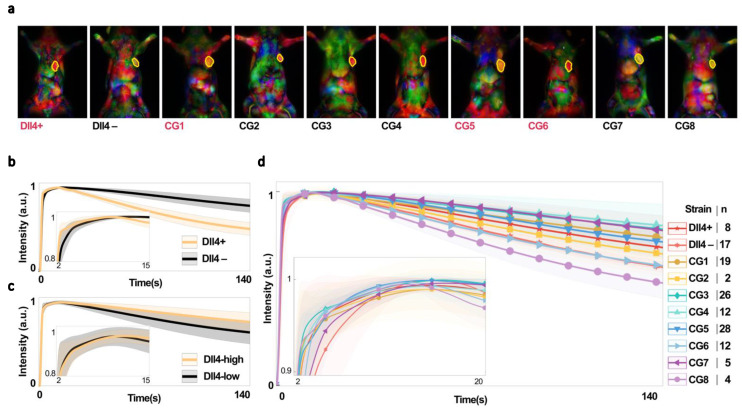
(**a**) Color-coded and merged principal component images were used to anatomically segment tumors from a single view/projection for rat strains with high (red) or low (black) levels of Dll4 expression. The top 3 ranked PCs for detecting tumors are merged as red, green, and blue channels of a true color (RGB) image. Tumors are outlined in yellow. (**b**–**d**) Mean intensity of ICG biodistribution fluorescence kinetic data over 140 s for (**b**) parental (Dll4+) and consomic (Dll4−) rats, (**c**) all Dll4−high and Dll4−low rats, and (**d**) all strains, plotted and normalized to a range of 0 to 1. Shaded regions show s.e.m.

Our study also implemented a novel method of ranking PCA components based on the 2D cross-correlation of a reference image containing the tumor. This added to the simplicity and computational efficiency of the framework. However, it should be noted that this method may not be effective for detecting tumors with random locations. On the other hand, it could be useful for detecting tumors or tissues of interest with high localization, such as the lungs, liver, and kidney, and lesions in breast tissue. Overall, our method shows potential for improving the accuracy and efficiency of tumor detection using NIR imaging and PCA (Figure 4a). However, further experimentation is needed to expand the framework to a general tumor detection algorithm.

### 4.2. Dll4 and Its Effect on the NIR Time Series

The analysis of the average fluorescence intensity of indocyanine green (ICG) in the tumor tissue of Dll4+ and Dll4− rats bearing triple-negative breast cancer (TNBC) tumors revealed that ICG uptake occurred more rapidly in Dll4− tissues and was retained for longer periods of time compared to Dll4+ hosts (Figure 4b). This indicates systemic differences in vascular function between the two rat strains. Our previous histological data showed that Dll4+ tumors have a higher vascular density and tortuosity, indicating a genetic microenvironment that promotes nonproductive angiogenesis [38]. This is further supported by the slower ICG wash-out observed in the Dll4+ tumors. These findings provide insight into the effects of host genetics on tumor angiogenesis and suggest potential therapeutic targets for TNBC.

In order to further investigate the role of Dll4 in vascular function in tumors, we divided chromosome 3 into regions with and without the Dll4 gene in congenic rat strains (Figure 1b) and then examined the ICG fluorescence intensity of tumors in Dll4-high and Dll4-low rats (Video S2) (Figure 4c). Our findings reveal significant systemic differences in vascular function between tumors in Dll4+ and Dll4− rats (parental strains), indicating the critical role of the Dll4 gene in tumor angiogenic response [38]. However, analysis of the ICG fluorescence intensity of tumors for individual strains (Figure 4d) reveals more complex behavior than the obvious differences in wash-in and wash-out patterns observed between Dll4+ and Dll4−. This supports the need for further investigation into the impact of Dll4 on NIR time series signatures and the potential use of Dll4-directed therapies for cancer treatment.

It is worth noting that although there are significant differences in Dll4-low vs. Dll4-high rat strains (when all the strains of Dll4 expression levels are combined), they are inconsistent with the observations made in Dll4+ and Dll4− rats. These results have significant implications for developing novel therapies that target Dll4 and other host TME modifiers involved in angiogenesis, as they demonstrate the critical role of these genes in tumor vascular function and angiogenic response.

Additionally, our research further highlights the capricious nature of the NIR signal, which is influenced by various heritable tumor microenvironments across different groups, as shown in Figure 4b,c. We aim to illustrate and categorize the impact of the Dll4 expression level on the NIR signal through this erratic behavior.

**Figure 5:**
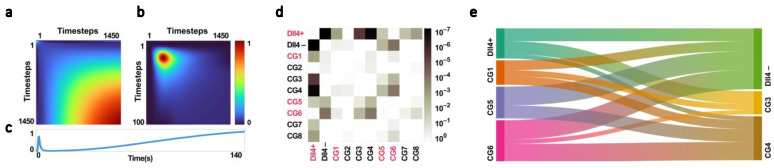
(**a**,**b**) The covariance parameter of a mixed-effects model for 1400 timesteps with appropriate time-varying covariates, used to analyze the average fluorescence intensity of ICG in the tumor with multiple measurements per subject, with the subject number serving as the repeated measure indicator and the rat strain serving as a covariate. (**c**) Projection of the diagonal of the covariance parameter on the time axis. (**d**) Multiplicity-adjusted *p*-value matrix showing significant differences between groups based on multiple comparison of estimated marginal means using the Tukey–Kramer test statistic. (**e**) Sankey diagram showing differences in strains with high and low levels of Dll4 expression based on the *A_score_* (mean of accuracy, sensitivity, and specificity in the primary classification of each pair); 0.65 was subtracted from all the *A_score_* values for better visualizations of differences.

We used a repeated measures model to analyze the average fluorescence intensity of ICG in the tumor over time, with the rat strain serving as a covariate. Figure 5a,b show the estimated response covariances matrix, which is the covariance of the repeated measures. The higher values in this matrix indicate the time points at which groups experience the greatest differences. By projecting the diagonal of the covariance matrix onto the time axis (Figure 5c), we were able to visualize the amount of difference between groups over time.

This projection, when compared to the average fluorescence intensity of ICG in the tumor (Figure 4b,d), showed the strongest differences between groups at the points where the NIR signal regions from half of its peak value to the peak value and at the tail of the curve, which are measures of the temporal inhomogeneity of the initial uptake and the decay of ICG fluorescence, were found to be particularly useful in discriminating between groups with different levels of Dll4 expression. This projection of the diagonal of the estimated response covariances matrix on the time curve can be used in feature design to focus on regions with the maximum amount of useful information for discriminating between groups and, subsequently, between classes with different levels of Dll4 expression. This could potentially improve the accuracy of tumor classification and ultimately improve therapy outcomes.

Our repeated measures model, which included responses as measurements and strains as predictor variables, allowed us to conduct multiple comparisons of estimated marginal means between groups. The resulting *p*-value matrix (Figure 5d) revealed significant differences in estimated marginal means between the Dll4+ and Dll4− groups, with a *p*-value of 4.71 × 10^−7^. In addition, we observed significant differences between Dll4+ and CG3, CG4, and CG8, with *p*-values of 1.67 × 10^−5^, 7.03 × 10^−7^, and 2.18 × 10^−3^, respectively.

For each group pair with high and low Dll4 expression levels, the separation score was calculated. First each of Dll4+|CG#−, CG#+|Dll4−, and CG#+|CG#− went through our classification algorithm with 10-fold cross-validation using Nearest Neighborhood, Linear SVM, RBF SVM, Decision Tree, Naive Bayes, and Logistic Regression models. The highest average classification metrics (Accuracy + Specificity + Sensitivity)/3 for Dll4+|CG#−, CG#+|Dll4− and CG#+|CG#−) was used to calculate the separation score (Score Dll4+|CG#− + Score CG#+|Dll4− + 2 × Score CG#+|CG#−)/4.

Furthermore, our analysis showed significant differences between Dll4− and CG5 and CG6, with *p*-values of 8.70 × 10^−3^ and 2.58 × 10^−4^, respectively. This supports the hypothesis that Dll4 expression levels can act as a heritable TME modifier on NIR time series intensity. However, the smallest *p*-value between Dll4+ and Dll4− suggests that there are other factors on chromosome 3, in addition to Dll4, that contribute to the observed differences in the NIR time series signature between these groups.

In contrast, no significant differences were found between Dll4− and the congenic strains with low levels of Dll4 expression (CG2, CG3, CG4, CG7, and CG8). This further supports the notion that Dll4 plays a crucial role in determining tumor vascular function and NIR time series intensity.

Among the congenic groups, the most significant differences were observed between CG5, CG6, and CG3, CG4 from the Dll4-high and Dll4-low groups, respectively. Notably, the differences were most significant between CG4 and CG6, with a *p*-value of 0.0003. This suggests that very narrow regions of differences on chromosome 3 between these two groups, one containing Dll4 and the other lacking it, have a significant effect on the NIR time series signature.

### 4.3. Primary Classification and Congenic Dissimilarity

The relationship between Dll4 expression and classification performance was analyzed using a total of 12 congenic pairs with *n* > 5 based on their levels of Dll4 expression (Dll4+|Dll4−, Dll4+|CG3, Dll4+|CG4, Dll4−|CG1, Dll4−|CG5, Dll4−|CG6, CG1|CG3, CG1|CG4, CG3|CG5, CG3|CG6, CG4|CG5, and CG4|CG6). The pairs were then subjected to a primary classification algorithm and their mean performance metrics, the *A_score_*, were calculated and reported in Table 1. The congenic pairs with low levels of Dll4 expression showed a mean *A_score_* of 0.91 +/− 0.01, indicating a high level of classification performance when compared to the Dll4+ parental strain. In contrast, the congenic pairs with high levels of Dll4 expression showed a mean *A_score_* of 0.8 +/− 0.05 when classified against the Dll4− consomic strain. Among the congenic pairs, the CG5|CG4 pair demonstrated the highest *A_score_* of 0.8, followed by the CG6|CG4 and CG6|CG3 pairs with *A_score_* values of 0.78 and 0.77, respectively. The results of the *A_score_* calculation are visualized in Figure 5e through a Sankey diagram.

To account for potential differences between the congenic pairs and the parental pairs, the *S_score_* was calculated. The CG5|CG4, CG6|CG3, and CG6|CG4 pairs showed the highest *S_score_* values of 0.84, 0.84, and 0.85, respectively, and were selected for the feature selection step. These results align with the multiple comparison of estimated marginal means between groups, indicating that CG5|CG4, CG6|CG3, and CG6|CG4 show the strongest differences in classification performance.

### 4.4. Feature Selection

RFE is a wrapper method that evaluates the entire classification algorithm and has shown improved classification accuracy and reduced overfitting compared to other feature selection methods [69]. However, RFE can be sensitive to noise and irrelevant features, leading to suboptimal feature subsets and reduced classification performance. Additionally, RFE is computationally intensive, which can pose a challenge for large datasets with a high number of features. Despite these limitations, RFE remains a valuable tool for selecting an optimal subset of features that maximizes classification performance [70,71].

To address these limitations, we performed feature selection in two steps to optimize the selection process and improve the performance of the classifier. First, we used RFE to select only two features out of the 86 available features for congenic pairs Dll4+|Dll4−, Dll4+|CG4, CG5|Dll4−, CG5|CG4, CG6|Dll4−, and CG6|CG4. The CG3 and its combinations (CG6|CG3, CG5|CG3, and Dll4+|CG3) were dropped from the feature selection process as the performances of the classifiers, the *A_score_*, using only two features were below 0.70, and lower than the other strains. The congenic pairs CG5|CG4 and CG6|CG4 as well as the parental and consomic group Dll4+|Dll4− went through our feature selection algorithm, and for each pair the two best-performing classification algorithms based on *A_score_* and associated feature pair were reported (Table 2).

The highest *A_score_* for the two best-performing models for CG5|CG4 was 0.78 ± 0.04 compared to CG6|CG4 with an *A_score_* of 0.72 ± 0.19 and 0.72 ± 0.22, resulting in the selection of CG5|CG4 for final congenic pair selection.

Finally, from each pair of Dll4+|Dll4−, Dll4+|CG4, CG5|Dll4, and CG4|CG5, four of the best performing features regardless of the ML model were chosen and were used as a collection of features for the final feature selection (Table 2). A combination of parental and consomic groups and the final selected congenic pair (Dll4+, Dll4−, CG5, and CG4) was used to select the final feature pair out of the 16 selected features, resulting in the selection of HIF5_avg and HIF50_avg as the best-performing features.

### 4.5. Performance of the Classification Models Based on the Selected Features

**Figure 6:**
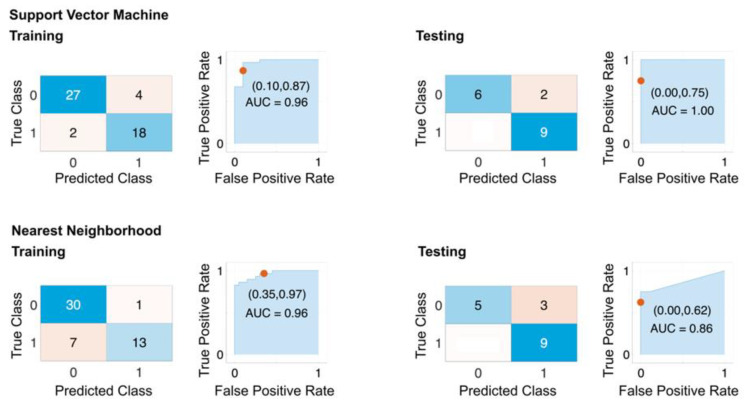
Performance of selected features on original dataset. The confusion matrix and ROC plots for training and testing of best-performing models based on HIF5_avg and HIF50_avg features with 10-fold cross-validation. The dataset includes all rat strains except Dll4+, Dll4−, CG4−, and CG5+. An amount of 25% of the dataset was used for holdout testing. Top row: SVM model. Bottom row: Nearest Neighborhood.

To evaluate the performance of the selected features, we trained datasets consisting of all the remaining congenic groups excluding the Dll4+, Dll4−, CG4, and CG5 (CG1 to CG3 and CG5 to CG8) using 10-fold cross-validation and keeping 25% of the dataset for testing the trained models. This allowed us to assess the generalizability of our model and test it on previously unseen datasets. The results of this step are reported as a confusion matrix, ROC curve, and AUC (Figure 6), as well as general classification metrics (Table 3). The best-performing models based on the selected features were SVM and KNN, with sensitivity and specificity of 1.00 and 0.81 and 1.00 and 0.75, respectively.

**Figure 7:**
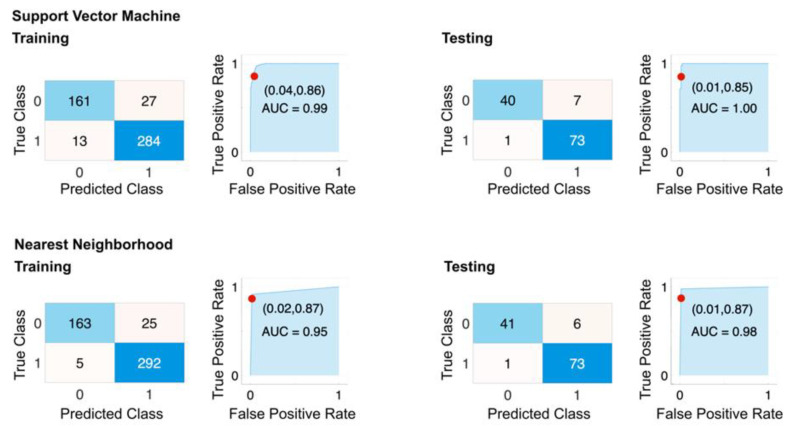
Performance of selected features on augmented dataset. The confusion matrix and ROC plots for training and testing of best-performing models based on HIF5_avg and HIF50_avg features with 10-fold cross-validation. The dataset includes all rat strains except Dll4+, Dll4−, CG4−, and CG5+. 25% of the dataset was used for holdout testing. Top row: SVM model. Bottom row: Nearest Neighborhood.

In order to further assess the effectiveness of our model, the selected features, and the generated congenic pair, we generated an augmented dataset consisting of all remaining congenic pairs excluding Dll4+, Dll4−, CG4 and CG5 (CG1 to CG3 and CG5 to CG8, with random variations in rotation, horizontal flip, and limited scaling (up to ±2%) to increase the diversity of the dataset. This resulted in a total of 606 data points. The performance of the models was evaluated using 10-fold cross-validation and a 20% hold out. The results of this step were reported as a confusion matrix, ROC curve, AUC (Figure 7), and overall classification metrics (Table 4). The best-performing models based on the selected features were SVM and KNN, with sensitivity and specificity of 0.97 and 0.91, and 0.97 and 0.92, respectively. These results align closely with the performance of the models over the original dataset, indicating the generalizability of our framework.

It is noteworthy that of the 16 most contributing features used to select the final feature pair, 12 were the newly proposed HIF features, and the other 4 were DS features, which we previously reported [48]. Additionally, the HIF features were amongst the best features for identifying genetic TME modifiers.

The relationship between covariance of the repeated measures and optimal feature design in machine learning classification algorithms is an essential factor in developing effective classification algorithms. Combined with our recent report [47], our analysis found that the DS and HIF features, which are generated in regions where the NIR signal varies from half of its peak value to the peak value and at the tail of the curve, were particularly effective in discriminating between benign/malignant tumors (DS features) and groups with different levels of Dll4 expression (HIF features). Furthermore, the projection of the covariance matrix onto the time axis revealed similar regions, indicating a relationship between this projection and optimal feature design. These findings have significant implications for feature design in machine learning classification algorithms. By focusing on the regions with the greatest amount of useful information for discrimination, we can design features specifically to capture these differences and improve the accuracy of tumor classification. This can ultimately lead to better therapy outcomes for patients. It is worth noting that this relationship between the covariance of the repeated measures and optimal feature design is not limited to HIF features and the specific context of our analysis. In general, considering the covariance of repeated measures can provide valuable information for identifying key regions and designing effective features for machine learning classification algorithms.

## 5. Conclusions

Dynamic vascular imaging techniques such as DCE-MRI and perfusion CT are used to extract multiple vascular parameters and have been used in clinical trials of anti-angiogenic drugs. However, these techniques have limitations, such as low temporal resolution and the need for specialized hardware and software [41,42]. To overcome these limitations, dynamic near-infrared (NIR) fluorescence imaging can serve as an effective alternative for characterizing germline-dependent vascular phenotypes. It can be combined with other modalities, such as in a paired-agent or multimodal MRI and fluorescence tomography approaches for noninvasive quantification of response to anti-angiogenesis therapy and classifying in vivo vascular phenotypes [7,43,44,67]. Furthermore, DE-NIR imaging, as a potential alternative for characterizing germline-dependent vascular phenotypes in preclinical models, can be extended to clinical modalities upon validation with cross-sectional dynamic contrast enhanced imaging.

The present study proposes that by combining DE-NIR imaging and machine learning algorithms with consomic xenograft models with human tumors, the role of inherited notch protein Dll4 (rat variant of delta like canonical ligand 4) expression specifically in the host vascular microenvironment can be studied. Specifically, in the context of breast cancer, where different genetic subtypes can impact treatment outcomes, identifying patients with high or low DLL4 (human variant of delta like canonical ligand 4) expression levels through noninvasive imaging could assist in selecting personalized treatment options. Nonetheless, the study authors acknowledge notable differences between the rat model utilized in the study and the human system, which could affect the generalization of the findings to human cases, as in human tumors DLL4 expression maybe be both on tumor cells and host vasculature, whereas in our CXM model, we focused specifically on the inherited variation in rat-derived host vasculature Dll4 expression in human xenograft tumors. Such differences may result in amplification or suppression of vascular phenotype responses if both tumor cells and the host microenvironment express high levels or contrasting levels of DLL4. However, even in that case, dynamic imaging will be useful in identifying patients likely to respond better to DLL4 targeted therapies.

Future studies will be necessary to validate this study’s findings, to assess the reliability and validity of the developed imaging and machine learning algorithms in a large and diverse patient population to determine if contrast agent kinetic profiles observed in human DCE-MRI or dyna-CT imaging datasets for primary and/or metastatic disease differ in human patients with high vs. low DLL4 expression. In the metastatic setting, where surgery is no longer an option, a machine-learning-enabled analysis of dynamic contrast-enhanced imaging will be valuable to assess the expression levels of DLL4 and guide therapy selection, especially in cases where a biopsy is not taken or if biopsy results are inconclusive [72,73]. Human anti-DLL4 antibodies have been reported for cancer treatment [54,74,75,76]. In one study, immunotoxin DLL4Nb-PE was developed, potentially as a cell cytotoxic agent and angiogenesis maturation inhibitor [76]. Another study successfully developed a bispecific monoclonal antibody that targets both human DLL4 and VEGF and showed efficacy in inhibiting proliferation, migration, and tube formation of human umbilical vein endothelial cells (HUVEC) [74]. In a phase 1a trial, navicixizumab, a bispecific antibody that inhibits DLL4 and VEGF, was tested in refractory solid tumor patients and showed the potential to inhibit tumor growth [75]. While DLL4 blockade is an attractive therapy, long-term extended use of DLL4 mAbs has demonstrated concerning off-target effects [77,78]. Pharmacokinetic modulation of DLL4 mAbs may reduce off-target effects [77], such as via short-term administration or by focusing on patients where dynamic contrast imaging indicates a high DLL4 vascular phenotype. As we have shown in prior work, high vascular DLL4 expressing tumors may also be susceptible to DLL4 targeted nanomedicine [38] or as combination therapy with anti-DLL4 monoclonal antibodies with nanomedicine drugs such as nab-paclitaxel (Abraxane) or Liposomal Doxorubicin (Doxil^TM^).

The use of noninvasive DE-NIR imaging to detect heritable TME modifiers is significant for several reasons. First, this method allows for the identification of potential modifiers without the need for invasive procedures, reducing the potential for discomfort and complications for patients. Second, the use of machine learning and DE-NIR imaging to develop a predictive model for cancer nanomedicine therapy can support effective decision making in the treatment process. While data processing and preparation and algorithm training can be complex, the resulting algorithms are simple and allow for the prediction of heterogeneity in a single step using ROI brightness measurements. Interestingly, traditional features such as time-to-peak and upslope do not appear in our selection of the most discriminative features. However, two novel features derived from HIF (HIF5_avg and HIF50_avg), which is a measure of the temporal inhomogeneity of both the initial uptake and decay of ICG fluorescence, were identified.

It is important to note that the training and testing sets used in this study are minimal, and therefore the high accuracy rates obtained should be interpreted with caution. Further research with larger datasets will be necessary to assess the reliability and validity of these findings with confidence.

We have reported novel dynamic enhanced near-infrared (NIR) fluorescence imaging and machine learning algorithms to noninvasively assess Dll4 expression levels in tumors. Our results showed that observation of subtle differences in vasculature structure and perfusion patterns characterized by ICG time kinetics could be used to differentiate between inherited tumor vascular microenvironment differences, such as Dll4 expression levels. Additionally, our analysis demonstrated the importance of considering the covariance of the repeated measures in the design of features for machine learning classification algorithms. By utilizing this information, we can improve the accuracy of tumor classification and ultimately improve therapy outcomes for patients.

To summarize, based on our recent study, we investigated the impact of genetically heterogeneous notch-Dll4 inheritance on the contrast agent uptake and clearance in triple-negative breast cancer xenografts. The differences in Dll4 inheritance have been shown to impact nanomedicine biodistribution, pharmacokinetics, and therapy response in our prior work. Thus, our results indicated that imaging can be potentially employed for selecting patients for Dll4-directed therapies by identify host microenvironments with high- or low-expressing Dll4 inheritance. This further suggests that the success of nanomedicine might depend on hereditary tumor microenvironment genes, regardless of tumor type. Additionally, host genes such as Dll4 can affect individual differences in NP uptake and response to NP-mediated therapies, providing the potential for more effective personalized Dll4 targeted nanomedicine for therapy-resistant hosts. Further studies are needed to validate these findings and explore the potential clinical applications of this approach.

## Figures and Tables

**Table 1:** Separation score for group pairs.

Groups	Average Classification Metrics (*A_score_*)			Separation Score (*S_score_*)
	Dll4+|CG−	CG+|Dll4−	CG+|CG−	
CG1|CG3	0.9	0.74	0.72	0.775
CG1|CG4	0.92	0.74	0.75	0.7875
CG5|CG3	0.9	0.83	0.61	0.7925
CG6|CG3	0.9	0.86	0.778	0.8495
CG5|CG4	0.92	0.83	0.8	0.845
CG6|CG4	0.92	0.86	0.78	0.855

**Table 2:** Primary validation and feature selection.

Groups	Feature	Best Average		Best Accuracy		Best Sensitivity		Best Specificity	
		Alg.	Value(std)	Alg.	Value(std)	Alg.	Value(std)	Alg.	Value(std)
Dll4+|Dll4−	HIF10_avg, HIF12_avg	DT	0.8666 (0.2309)	DT	1 (0)	DT	0.6 (0.5026)	DT	1 (0)
	HIF6_avg, HIF50_avg	LR	0.7597 (0.1412)	LR	0.8682 (0.1468)	LR	0.6 (0.5026)	DT	0.9391 (0.116)
Dll4+|CG3	HIF300_avg, HIF200_avg	RBF SVM	0.6415 (0.3196)	RBF SVM	0.5914 (0.0983)	L SVM	1 (0)	KNN	0.9166 (0.1147)
	HIF300_avg, TR_rel	LR	0.6453 (0.1036)	LR	0.6176 (0.1624)	LR	0.85 (0.2665)	KNN	0.9083 (0.1147)
Dll4+|CG4	HIF30_avg, D16_avg	DT	0.9175 (0.0303)	DT	0.9125 (0.0915)	L SVM	0.95 (0.0888)	NB	1 (0)
	HIF8_avg, D6_avg	RBF SVM	0.88125 (0.1254)	RBF SVM	0.89375 (0.1174)	L SVM	1 (0)	NB	0.85 (0.2016)
CG5|Dll4−	HIF5_avg, HIF16_avg	DT	0.8251 (0.0698)	DT	0.8292 (0.1093)	L SVM	1 (0)	NB	0.845 (0.1952)
	HIF8_avg, HIF25_avg	KNN	0.8044 (0.1505)	KNN	0.8201 (0.0919)	L SVM	1 (0)	NB	0.7925 (0.2014)
CG5|CG3	HIF300_avg, HIF400_avg	L SVM	0.5683 (0.4037)	LR	0.5292 (0.1507)	L SVM	1 (0)	KNN	0.85 (0.1613)
	HIF300_avg, HIF50_avg	LR	0.5919 (0.4037)	LR	0.5626 (0.1652)	L SVM	1 (0)	KNN	0.8333 (0.1324)
CG5|CG4	D18_avg, HIF4_rel	KNN	0.7811 (0.0485)	KNN	0.7948 (0)	L SVM	0.8928 (0)	DT	0.7272 (0)
	D18_avg, HIF4_avg	KNN	0.7811 (0.0485)	KNN	0.7948 (0)	L SVM	0.8928 (0)	DT	0.7272 (0)
CG6|Dll4−	D40_avg, D2_avg	KNN	0.8685 (0.0404)	KNN	0.8755 (0.1180)	L SVM	0.95 (0.1574	KNN	0.905 (0.1422)
	D40_avg, HIF5_rel	KNN	0.8482 (0.01263)	KNN	0.8440 (0.1386)	RBF SVM	0.9375 (0.1293)	KNN	0.8383 (0.1643)
CG6|CG3	HIF300_avg, HIF350_avg	L SVM	0.5729 (0.3995)	KNN	0.5318 (0.1215)	L SVM	1 (0)	NN	0.8583 (0.1733)
	HIF300_avg, HIF180_avg	RBF SVM	0.5890 (0.2399)	RBF SVM	0.5570 (0.1259)	L SVM	1 (0)	KNN	0.85 (0.2222)
CG6|CG4	HIF8_avg, HIF23_avg	KNN	0.7278 (0.1917)	KNN	0.7437 (0.0904)	RBF SVM	1 (0)	NB	0.7285 (0.2853)
	HIF6_avg, HIF50_avg	NB	0.7280 (0.2263)	NB	0.7468 (0.0982)	L SVM	1 (0)	DT	0.6071 (0.1846)

For each group pair with high and low Dll4 expression levels, 2 sets of feature pairs and best-performing algorithms listed. For each algorithm, accuracy, sensitivity, specificity metrics, and average metric (Accuracy + Specificity + Sensitivity)/3 shown. Alg.: classifier algorithm, KNN: Nearest Neighborhood, L SVM: Linear SVM, RBF SVM: Radial Basis Function kernel SVM, DT: Decision Tree, NB: Naive Bayes, LR: Logistic Regression models.

**Table 3:** Performance of selected features.

	SVM		KNN	
Measure	Training	Testing	Training	Testing
Sensitivity	0.9310	1.0000	0.8108	1.0000
Specificity	0.8182	0.8182	0.9286	0.7500
Precision	0.8710	0.7500	0.9677	0.6250
Negative Predictive Value	0.9000	1.0000	0.6500	1.0000
False-Positive Rate	0.1818	0.1818	0.0714	0.2500
False Discovery Rate	0.1290	0.2500	0.0323	0.3750
False-Negative Rate	0.0690	0.0000	0.1892	0.0000
Accuracy	0.8824	0.8824	0.8431	0.8235
F1 Score	0.9000	0.8571	0.8824	0.7692
Matthews Correlation Coefficient	0.7600	0.7833	0.6758	0.6847

The training and testing metrics of the best-performing models based on the selected features HIF5_avg and HIF50_avg with 10-fold cross-validation are shown. The training dataset includes features extracted from the original NIR time series data of all congenic groups except CG5+ and CG4−, and 25% of the dataset was used for holdout testing of the models. SVM: Support Vector Machine, KNN: Nearest Neighborhood.

**Table 4:** Performance of selected features on augmented dataset.

	SVM		KKN	
Measure	Training	Testing	Training	Testing
Sensitivity	0.9253	0.9756	0.9702	0.9762
Specificity	0.9132	0.9125	0.9211	0.9241
Precision	0.8564	0.8511	0.867	0.8723
Negative Predictive Value	0.9562	0.9865	0.9832	0.9865
False Positive Rate	0.0868	0.0875	0.0789	0.0759
False Discovery Rate	0.1436	0.1489	0.133	0.1277
False Negative Rate	0.0747	0.0244	0.0298	0.0238
Accuracy	0.9175	0.9339	0.9381	0.9421
F1 Score	0.8895	0.9091	0.9157	0.9213
Matthews Correlation Coefficient	0.8254	0.8625	0.8705	0.8793

The training and testing metrics of the best-performing models based on the selected features HIF5_avg and HIF50_avg with 10-fold cross-validation are shown. The training dataset includes features extracted from the original NIR time series data of all congenic groups except CG5+ and CG4-, and 25% of the dataset was used for holdout testing of the models. SVM: Support Vector Machine, KNN: Nearest Neighborhood.

## Data Availability

This study employed the established consomic rat models SS and SS.BN3 as well as our congenic strains CG1 to CG8. The publicly accessible and NIH-supported Rat Genome Database (rgd.mcw.edu) catalogs has tools to explore the genotype and phenotype information for the SS (Dll4+) and SS.BN3 (Dll4−) and congenic strains under strain records Dll4+ (RGDID:61499), Dll4− (RGDID:1358154), CG1 (RGDID:155782881), CG2 (RGDID:155782883), CG3 (RGDID:155782884), CG4 (RGDID:155791428), CG5 (RGDID:155791426), CG6 (RGDID:155791430), CG7 (RGDID:155791429), and CG8 (RGDID:155791427).

## Supplementary Materials

The following supporting information can be downloaded at: https://www.mdpi.com/article/10.3390/cancers15051460/s1, Figure S1: Principal component analysis; Video S1: RespMotionCorretion; Video S2: ICG_NIR_All_Strains.
