# Supplementary figures and images for: Dynamic NIR Fluorescence Imaging and Machine Learning Framework for Stratifying High vs. Low Notch-Dll4 Expressing Host Microenvironment in Triple-Negative Breast Cancer
Source: Cancers (Basel). 2023 Feb 25;15(5):1460. doi: 10.3390/cancers15051460 (PMC10000786; doi:10.3390/cancers15051460)

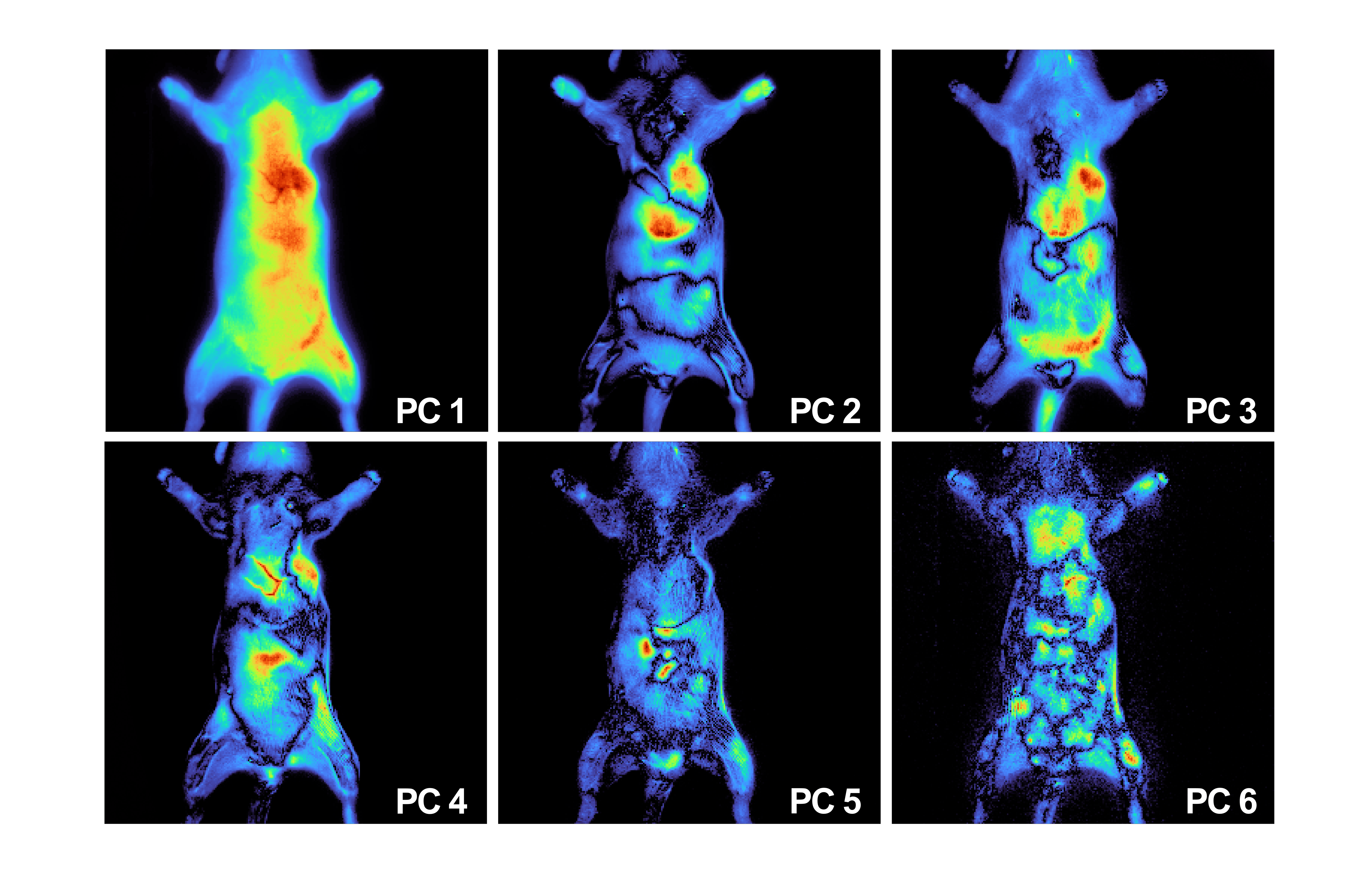

Supplement: Supplementary file 1 [file cancers-15-01460-s001.zip › Figure S1.tif]
